# Single Amino Acids G196 and R198 in hr1 of Subgroup K Avian Leukosis Virus Glycoprotein Are Critical for Tva Receptor Binding

**DOI:** 10.3389/fmicb.2020.596586

**Published:** 2020-12-16

**Authors:** Jian Chen, Jinqun Li, Lizhen Li, Peng Liu, Yong Xiang, Weisheng Cao

**Affiliations:** ^1^College of Veterinary Medicine, South China Agricultural University, Guangzhou, China; ^2^Guangdong Laboratory for Lingnan Modern Agriculture, Guangzhou, China; ^3^Key Laboratory of Zoonosis Prevention and Control of Guangdong Province, Guangzhou, China; ^4^Key Laboratory of Veterinary Vaccine Innovation of the Ministry of Agriculture, Guangzhou, China; ^5^National and Regional Joint Engineering Laboratory for Medicament of Zoonosis Prevention and Control, Guangzhou, China

**Keywords:** retrovirus, recombinant viruses, fluorescence-activated cell sorting, avian leukosis viruses, envelope glycoprotein

## Abstract

Avian leukosis viruses (ALVs), a type of retrovirus responsible for various tumor diseases in chickens, are divided into 11 subgroups: ALV-A to ALV-K. After the envelope glycoproteins of ALV interact with the cellular receptor to initiate viral invasion, alterations in a few amino acids of the viral glycoproteins or cell receptors may trigger changes in their conformation and binding affinity. To identify the functional determinants of the ALV-K envelope protein that binds to Tva (a recently identified cellular receptor of ALV-K), using the strategy of continuous, segment-by-segment substitution of the gp85-encoded surface glycoprotein (SU) of ALV-K GDFX0602 with ALV-E ev-1 (using Tvb as the receptor), a series of chimeric soluble gp85 proteins were expressed for co-immunoprecipitation (co-IP) analysis and a series of recombinant viruses with replication-competent avian retrovirus vectors containing Bryan polymerase (RCASBP) as their skeleton were created for transfecting to DF-1 cells and titer determination. The co-IP analysis, fluorescence-activated cell sorting, and virus titer measurements revealed that the substitution of residues 194–198, 206–216 of hr1, residues 251–256 between hr1 and hr2, and residues 269–280 of hr2 were identified to reduce the binding of gp85 to Tva. The substitution of residues 194–221 in hr1 nullified the infectiveness of these viruses, similar to the effect of single amino acid mutations in K251E and L252I located between hr1 and hr2; continuous amino acid mutations in hr2 could not produce the same effect despite reducing their infectiveness. Finally, single amino acid mutations G196A and R198H nearly abolished the binding of gp85 to Tva and nullified the infectiveness of these viruses to DF-1. This study paves the way for exploring the molecular mechanisms of the binding of Tva to ALV-K SU.

## Introduction

Avian leukosis virus (ALV), a member of the family Retroviridae and genus *Alpharetrovirus*, is a common avian retrovirus associated with neoplastic and immunosuppression diseases (Fadly and Smith, 1999). ALV isolates are divided into 11 subgroups (A to K) based on viral interference, host range, genomic structure, and differences in the antigenic structures of their envelope glycoproteins. Among them, ALV-K is an emerging subgroup that was recently isolated and identified in China (Cui et al., 2014; Zhao et al., 2018). Although most ALV-K isolates are weak in replication and pathogenicity because of their endogenous long terminal repeats (LTRs) (Li et al., 2016; Su et al., 2018a), recently discovered ones exhibit relatively strong pathogenicity (Su et al., 2018b).

For ALV to invade a host cell, its membrane protein first needs to bind to a cellular receptor (Smith and Cunningham, 2007; Koslová et al., 2018). The degree of viral invasion into cells depends on the strength of binding, which is crucial for viral infection. A recent study on the minimum functional domain of ALV receptors has confirmed that a few specific amino acids play a role in binding to ALV envelope proteins and in mediating viral infection (Rong and Bates, 1995a; Knauss and Young, 2002; Klucking and Young, 2004; Kucerová et al., 2013; Guan et al., 2017). However, under evolutionary pressure of a receptor competitor select, the structure of ALV envelope glycoproteins can evolve to use different cellular proteins as receptors (Taplitz and Coffin, 1997; Federspiel, 2019; Munguia and Federspiel, 2019).

Particularly, the envelope glycoprotein of ALV-K facilitates viral invasion by specifically recognizing and binding to Tva, similar to the binding action of the envelope glycoprotein of ALV-A (Pøikryl et al., 2019). This envelope glycoprotein is composed of the gp85-encoded surface glycoprotein (SU) and the gp37-encoded transmembrane glycoprotein (TM). In the different subgroups of ALV, Env is the most differentiated region, especially because of its gp85, whose sequence could be divided into three variable regions (vr1, vr2, and vr3) and two host range determinant regions (hr1 and hr2), and ALVs recognize cell receptors through the interaction of two SU regions, hr1 and hr2, with the host cell surface receptor (Dorner and Coffin, 1986; Bova et al., 1988; Holmen et al., 2001).

The DF-1 cell line used in this study is a continuous fibroblastic cell line derived from line 0 CEFs, which is not susceptible to endogenous virus ALV-E (Federspiel et al., 1991) but to exogenous virus ALV-K. Endogenous virus ALV-E ev-1 (GenBank: AY013303.1) and exogenous virus ALV-K GDFX0602 (GenBank: KP686143.1) show considerable homology (LTR: 98.5%; gp37: 98.9%; gp85: 87.9%) (Zhao et al., 2018), and the regions with considerable differences are primarily located in hr1 and hr2. Unlike ALV-K, ALV-E shares the Tvb-encoded tumor necrosis factor receptor with ALV-B/D as a receptor that is encoded by three alleles: tvb^*s*1^, tvb^*s*3^, and tvb^*st*^ (Adkins et al., 1997). Under the premise of high homology between ALV-K and ALV-E, their receptors involved in invasion are different; therefore, the differences between the binding sites of ALV-K and ALV-E would determine the mechanism of cell invasion. In other words, hr1 and hr2 could be the key regions for determining the infection of ALV to DF-1 cells.

Based on the aforementioned assumption, the purpose of our study was to reveal whether hr1 and hr2 present in SU affect the infection-causing ability of ALV-K in DF-1 cells and determine which amino acid residues directly interact with Tva.

## Materials and Methods

### Cell Cultures and Antibodies

293T cells were cultured in Dulbecco’s modified Eagle’s medium (DMEM; Gibco, Life Technologies, Carlsbad, CA, United States) supplemented with 10% fetal bovine serum (BioInd, United States) in the presence of 5% CO_2_ at 37°C. In contrast, DF-1 cells were grown in DMEM with 10% FBS (Gibco) in the presence of 5% CO_2_ at 39°C. The anti-HA tag was purchased from Thermo Scientific (Rockford, IL, United States), whereas the anti-flag M2 tag antibodies and the anti-GAPDH antibodies were purchased from Sigma (Sigma-Aldrich, St. Louis, MO, United States).

### Construction of Chimeric RCASBP Vector With GDFX0602 and ev-1

**FIGURE 1:**
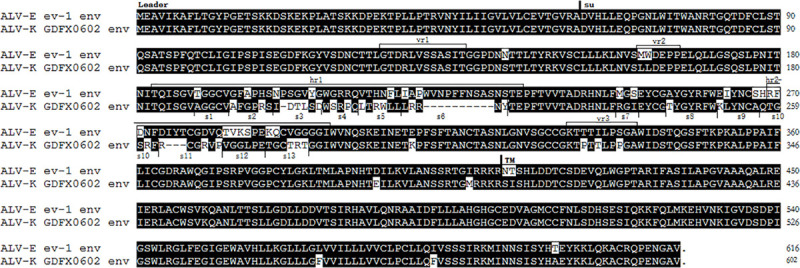
A comparison between the amino acids of ALV-K GDFX0602 and ALV-E ev-1 Env glycoproteins, with the envelope glycoprotein leader sequence (Leader), surface glycoprotein (SU) sequence, and transmembrane glycoprotein (TM) sequence indicated, along with the sequences of the variable regions (vr1, vr2, and vr3) and host range determinant regions (hr1 and hr2) in SU and TM.

Enhanced green fluorescent protein (EGFP) was amplified with EGFP-*Cla*I-F/R (Table 1) and inserted at the *Cla*I site of the replication-competent avian retrovirus vectors containing Bryan polymerase (RCASBP) vector, a replication-competent retroviral vector (RCAS) containing Bryan polymerase, according to the method of overlapping PCR for constructing the recombinant plasmid RCASBP (A)-EGFP. Next, RCASBP (A)-EGFP was digested with *Kpn*I and *Stu*I (New England Biolabs, Ipswich, MA, United States), and the fragment containing the 3′ end of *pol* and complete *env* was replaced with the 3′ end of *pol* and complete *env* from the GDFX0602 virus (Figure 1) for generating the newly constructed plasmid RCASBP (K)-EGFP.

**TABLE 1:** Primers used in this study.

**Primers^1^**	**Sequences 5′ to 3′**	**Length (bp)**
EGFP-*Cla*I-F	TACCACTGTGGCatcgatATGGTGAGCAAGG^2^	720
EGFP-*Cla*I-R	CCCGTACATCGCatcgatTTACTTGTACAGCTC^2^	
RCASBP-F	GGCAGGAAAGACAGCTATTGG	1,467
RCASBP-R	TGGCGACCACACCCGTCCTG	
F1	ACAGGGACACTGATAAGGTT	1,867
R1	CTTTCAGGCTGCCCACAGGCCTTTACACTGCTCCATTTTCGG	
RCASBP(K)-F	CTACCGTTCTTACAGAAGGACC	2,070
RCASBP(K)-R	CACACAGACAAAAGCGTATTTCAC	

*^1^F, forward primer; R, reverse primer. ^2^The lowercase letters were the sites for restriction digest.*

Construction of a series of chimeric viruses with reference to RCASBP (K)-EGFP is shown in Figure 1. Two domain-exchange chimeric RCASBP (K/E)-EGFP was constructed by replacing the residues of R1 motif (aa189–221, a part of hr1) and R2 motif (aa237–280, including hr2 and several residues between hr1 and hr2) of GDFX0602 with the corresponding sequences of ev-1 for every five or six amino acids (residues s1–s13) by overlapping PCR. Three single amino acid mutations of K251E, L252I, and A256S were also constructed by similar method.

### Expression of Various Chimeric Soluble gp85 Proteins

To identify the key amino acid residues of the ALV-K gp85 protein that interact with Tva, gp85 of ALV-K GDFX0602 was cloned into the eukaryotic expression vector pCAGGS and fused with the 3 × flag tag sequence. To ensure that the gp85 protein was expressed in a soluble form, a fragment encoding a signal peptide (pCAGGS-s-gp85-flag) was fused with the N terminus of the gp85 protein. Similar to the method of constructing RCASBP (K/E)-EGFP, a series of chimeric soluble gp85 proteins were constructed by replacing the corresponding sequence residues with the residues having maximum significant effect on ALV-K infection and the titers of GDFX0602 and ev-1 by overlapping PCR.

### Determination of GFP-Positive Cell Percentage and Viral Titer

CEFs, a primary cell line, is susceptible to both ALV-K and ALV-E, but it cannot be continuously viral passaged, so CEFs are not suitable for transfection of RCASBP(K)-EGFP series vectors to obtain sufficient virus. Therefore, DF-1 cells, a cell line that is not susceptible to ALV-E, were used as experimental materials to compare the replication ability of recombinant viruses. Recombinant RCASBP (K)-EGFP-based retrovirus vectors (0.5 μg) were transfected into DF-1 cells in a 12-well plate, and after three consecutive viral passages, one part of the cells was collected for determining the percentage of GFP-positive cells by fluorescence-activated cell sorting (FACS) using an LSRII analyzer (Becton, Dickinson). The other part of the cells was cultured in 1% FBS medium for 7 days, after which the supernatant was collected and diluted in gradient to determine the viral titer according to the Reed–Muench method.

### Co-immunoprecipitation Experiments and Pull-Down Assay

293T cells in 60-mm dishes were transfected with 5 μg of each respective chimeric gp85 plasmid using PolyJet (SignaGen Laboratories, Rockville, MD, United States) according to the manufacturer’s instructions. At 48 h after transfection, the supernatant of 293T cells was collected and filtered through a 0.22-μm filter membrane, followed by ultrafiltration to obtain a concentration of up to 1/10 of the volume for the next experiment. For the *in vitro* binding assay, Tva was fused with the human IgG-Fc fragment, which specifically bound to the protein A/G of the plasmid pCAGGS-Tva-HA-Fc that was expressed in 293T cells. The cell culture medium was collected, and the proteins were purified using protein A/G (Santa Cruz, Lexington, MA, United States) for 2 h at 4°C with gentle agitation. After five washes with ice-cold phosphate-buffered saline (PBS), the agarose was incubated with the cellular supernatant of 293T cells transfected with the respective recombinant pCAGGS-gp85-flag, and the respective gp85 was expressed for 6 h at 4°C with gentle agitation. After five washes with ice-cold PBS, the bound proteins were separated by SDS-PAGE, and western blotting was performed using mAbs against Tva-HA, gp85-flag, and GAPDH.

### Western Blotting

High-temperature-denatured proteins were separated on 12% SDS-PAGE gels and transferred onto a nitrocellulose membrane. After blocking with 5% (*w*/*v*) skimmed milk at room temperature for 1 h, the membrane was incubated with anti-flag mAb or anti-HA mAb at 4°C overnight. After three washes with PBS, the membrane was incubated with IRDye 680RD donkey anti-mouse IgG (H + L) antibodies (LI-COR Biosciences, Lincoln, NE) for 1 h at room temperature. Finally, the membrane blots were scanned using an Odyssey Infrared Imaging System (LI-COR Biosciences).

## Results

### Low Homology Between ALV-K GDFX0602 and ALV-E ev-1 of hr1 and hr2

**FIGURE 2:**
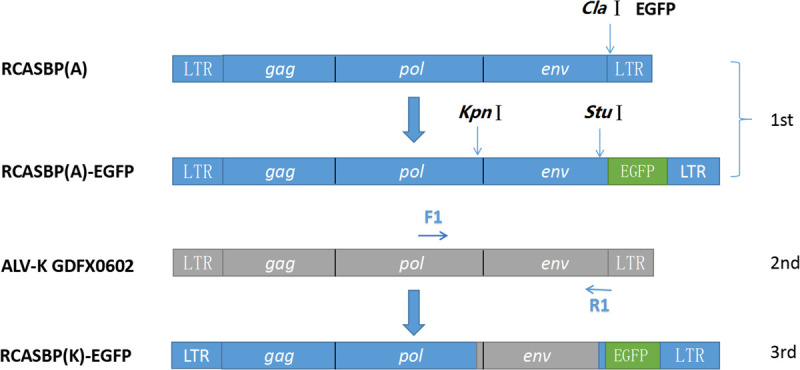
Strategy for constructing replication-competent avian retrovirus vectors containing Bryan polymerase (RCASBP). RCASBP (K)-enhanced green fluorescent protein (EGFP) recombinant virus EGFP was used with EGFP-*Cla*I-F/R and inserted at *ClaI* site of vector RCASBP. Primers RCASBP-F/R were used to validate the sequencing of vector RCASBP (A)-EGFP; primers F1 and R1 were used to amplify the Env fragments of the ALV-K GDFX0602 isolate, which was used to replace *env* of RCASBP (A)-EGFP; and primers RCASBP (K)-F/R were used to validate the sequencing of vector RCASBP (K)-EGFP.

The envelope glycoproteins of ALV-K GDFX0602 and ALV-E ev-1 showed major amino acid changes in hr1 and hr2. Variable regions were stable with change of only two amino acids in vr2 region, change of three amino acids in the vr3 region, and no change in hr1 (Figure 2). The homology between SU domains R1 (aa189–221, a part of hr1) and R2 (aa237–280, including hr2 and several residues between hr1 and hr2) of ALV-K GDFX0602 and ev-1 was low. The domains R1 and R2 have been divided into 13 segments (s1–s13) for subsequent research (Figure 2).

### Substitution of Domains R1 or R2 of ALV-K GDFX0602 With ALV-E ev-1 Rendered Recombinant ALV-K Non-infective in DF-1 Cells

**FIGURE 3:**
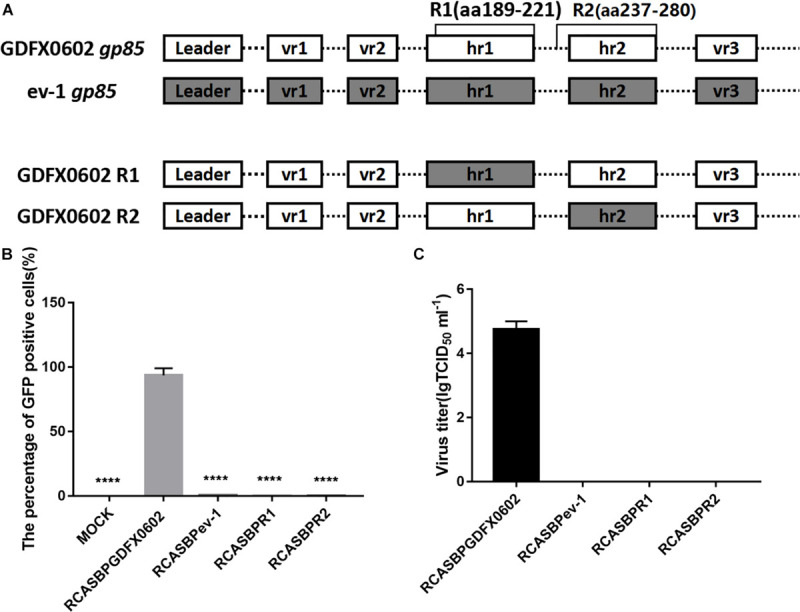
Both residues R1 (aa189–221) and R2 (aa237–280) affected the ability of ALV-K to infect DF-1 cells. **(A)** The relationship between R1, R2, and regions hr1 and hr2. **(B)** DF-1 cells transfected with recombinant plasmids RCASBPGDFX0602-enhanced green fluorescent protein (EGFP), RCASBPev-1-EGFP, RCASBPR1-EGFP, and RCASBPR2-EGFP were detected for the percentage of GFP-positive cells by fluorescence-activated cell sorting. **(C)** The supernatant was collected and diluted in gradient to determine the virus titers. Three independent experiments were performed, and data are shown as mean ± SD in triplicate from a representative experiment. Statistical analysis (two-way analysis of variance) was performed using GraphPad Prism 7; ****P < 0.0001.

To determine the role of R1 and R2 in the replication of ALV-K, the recombinant plasmids RCASBPR1-EGFP and RCASBPR2-EGFP, both based on the RCASBP vector, were used for transfection into DF-1 cells (Figure 3A). After three consecutive viral passages, the cells were collected to determine the percentage of GFP-positive cells by FACS. GFP-positive cells were barely detectable in RCASBPR1-EGFP- and RCABPR2-EGFP-transfected DF-1 cells, which was similar to the results of DF-1 cells (negative control) and RCASBPev-1-EGFP (Figure 3B). As expected, the percentage of GFP-positive cells in RCASBP GDFX0602-EGFP-transfected cells was nearly 100%. The results of virus titer obtained from cell supernatant were consistent with those obtained by FACS. The supernatant obtained from the cells transfected with recombinant plasmid RCASBPGDFX0602-EGFP had virus titer exceeding 10^4^ TCID_50_/ml, whereas the supernatant from cells transfected with recombinant plasmids RCASBPev-1-EGFP, RCASBPR1-EGFP, and RCASBPR2-EGFP produced no virus titers (Figure 3C).

### Residues 194–199 and 206–216 in hr1of ALV-K gp85 Were the Key Residues Binding to Tva

**FIGURE 4:**
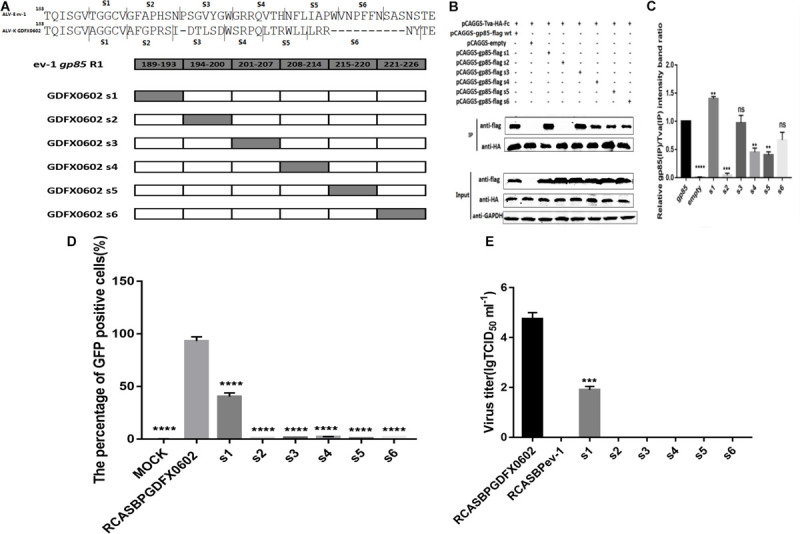
Residues 194–199 and 206–216 in hr1of ALV-K gp85 were the key residues binding to Tva. **(A)** The relationship between residues R1 (aa189–221) and s1–s6. **(B)** The interaction of soluble chimeric proteins gp85-flag s1–s6 with Tva-HA-Fc. **(C)** The level of protein was quantified using Image StudioLiteVer 5.2, and soluble chimeric proteins gp85-flag (IP)/Tva-HA-Fc (IP) were calculated. **(D)** DF-1 cells transfected with recombinant plasmids RCASBPGDFX0602 s1–s6-EGFP were detected for the percentage of GFP-positive cells by fluorescence-activated cell sorting. **(E)** The supernatant was collected and diluted in gradient to determine the virus titers. Three independent experiments were performed, and data are shown as mean ± SD in triplicate from a representative experiment. Statistical analysis (two-way analysis of variance) was performed using GraphPad Prism 7; **P < 0.01, ***P < 0.001, ****P < 0.0001.

To identify which amino acid residues are involved in the binding of gp85 to Tva, equal amounts of various chimeric soluble gp85 proteins (s1–s6) were used for co-immunoprecipitation (co-IP) (Figure 4A). The gray values of recombinant gp85 proteins s2, s4, and s5 were significantly lower than those of other recombinant gp85 proteins (*P* < 0.01), especially s2 (residues 194–199) of recombinant gp85, with almost no gray signals detected (Figures 4B,C, *P* < 0.001). For further verification, DF-1 cells transfected with recombinant plasmid RCASBPs1–s6-EGFP (Figure 4D) were collected after three consecutive viral passages to determine the percentage of GFP-positive cells by FACS. Except for RCASBPs1-EGFP, no GFP-positive cells were detected in any cells transfected by other recombinant plasmids. Moreover, the percentage of GFP-positive cells transfected with the plasmid RCASBPs1-EGFP was lower than that of RCASBP GDFX0602-EGFP (Figure 4D). RCASBPs1-EGFP could reproduce virus titer close to 10^2^ TCID_50_/ml, which was lower than that of RCASBP GDFX0602-EGFP (Figure 4E), and these results were consistent with the FACS. The titer results of the recombinant virus to some extent were coincided with the results of the protein interactions.

### Residues 251–256 Between hr1 and hr2, and Residues 269–280 in hr2 of ALV-K gp85 Were the Key Residues Binding to Tva

**FIGURE 5:**
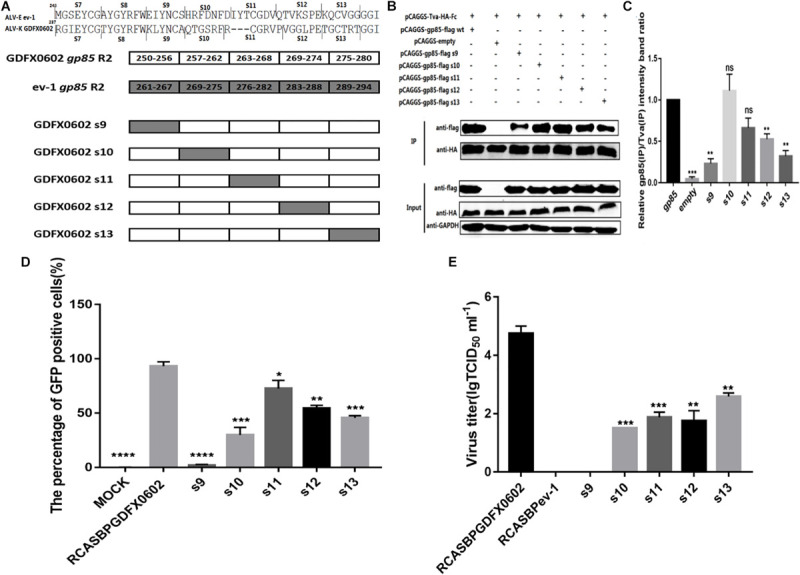
Residues 251–256 between hr1 and hr2, and residues 269–280 in hr2 of ALV-K gp85 were the key residues binding to Tva. **(A)** The relationship between residues R2 (aa237–280) and s7–s13. **(B)** The interaction of soluble chimeric proteins gp85-flag s9–s13 with Tva-HA-Fc. **(C)** The level of protein was quantified using Image StudioLiteVer 5.2, and soluble chimeric proteins gp85-flag (IP)/Tva-HA-Fc (IP) were calculated. **(D)** DF-1 cells transfected with recombinant plasmids RCASBP GDFX0602 s9–s13-EGFP were detected for the percentage of GFP-positive cells by fluorescence-activated cell sorting. **(E)** The supernatant was collected and diluted in gradient to determine the virus titers. Three independent experiments were performed, and data are shown as mean ± SD in triplicate from a representative experiment. Statistical analysis (two-way analysis of variance) was performed using GraphPad Prism 7; *P < 0.05, **P < 0.01, ***P < 0.001, ****P < 0.0001.

To further investigate the effects of hr2 on the binding affinity of recombinant gp85 protein to Tva, various chimeric soluble gp85 proteins (s9–s13) were used for co-IP (Figure 5A). The gray values of recombinant gp85 proteins s9, s12, and s13 were significantly lower than those of other recombinant gp85 proteins (Figures 5B,C, *P* < 0.01). The DF-1 cells transfected with recombinant plasmid RCASBP s9–s13 (Figure 5D) were collected after three consecutive viral passages to determine the percentage of GFP-positive cells by FACS. All s9–s13 residues decreased the percentage of GFP-positive cells, and residues s9 (aa251–256) showed maximum reduction in the percentage of GFP-positive cells (Figure 5D). The cell supernatants collected from the cells transfected with recombinant plasmid RCASBP s9-EGFP produced no virus titers (Figure 5E), and these findings were consistent with those obtained by FACS analysis. Moreover, the percentages of GFP-positive cells and virus titers of residues s10-, s11-, s12-, and s13-related RCASBP vector were significantly lower (*P* < 0.05) than those of RCASBP GDFX0602-EGFP (Figures 5D,E), which were basically consistent with the results of protein interactions.

### Single Amino Acids K251 and L252 Played a Decisive Role in the Replication of ALV-K *in vitro*

**FIGURE 6:**
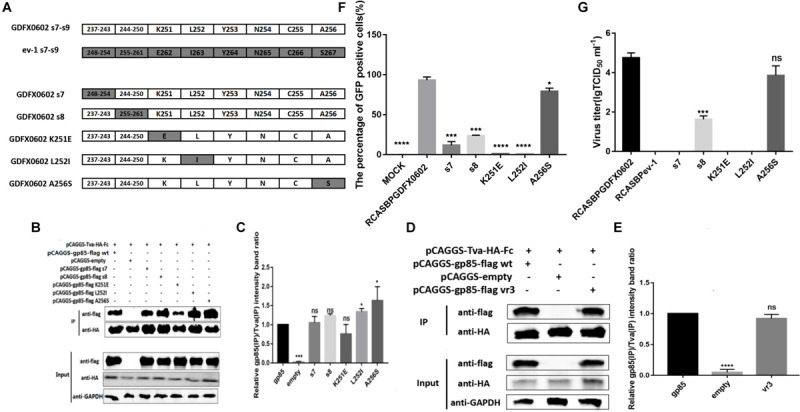
Residues K251 and L252 played a decisive role in the replication of ALV-K *in vitro*. **(A)** K251, L252, and A256 of GDFX0602 gp85 were replaced with ev-1 gp85. **(B,D)** The interaction of soluble chimeric proteins gp85-flag s7, s8, K251E, L252I, A256S and vr3 with Tva-HA-Fc. **(C,E)** The level of protein was quantified using Image StudioLiteVer 5.2, and soluble chimeric proteins gp85-flag (IP)/Tva-HA-Fc (IP) were calculated. **(F)** DF-1 cells transfected with recombinant plasmids RCASBP GDFX0602 s7-EGFP, RCASBP GDFX0602 s8-EGFP, RCASBP GDFX0602 K251E-EGFP, RCASBP GDFX0602 L252I-EGFP, and RCASBPGDFX0602 A256S-EGFP were detected for the percentage of GFP-positive cells by fluorescence-activated cell sorting. **(G)** The supernatant was collected and diluted in gradient to determine the virus titers. Three independent experiments were performed, and data are shown as mean ± SD in triplicate from a representative experiment. Statistical analysis (two-way analysis of variance) was performed using GraphPad Prism 7; *P < 0.05, ***P < 0.001, ****P < 0.0001, ^*ns*^*P* > 0.05.

Amino acid residue s9 at positions 251–256 of SU significantly affected the percentage of GFP-positive cells and virus titer of RCASBP-EGFP-related vectors (*P* < 0.0001). Single amino acids K251, L252, and A256 were identified as the key amino acids that were different between ALV-K gp85 and ALV-E gp85 at 251–256 positions (Figure 6A). The western blot gray levels of mutations K251E combined with Tva protein showed slightly lower than those of other recombinant gp85 proteins; however, there were no significant difference in protein levels (Figures 6B,C, *P* > 0.05). The mutations L252I and A256S significantly enhanced the binding affinity of gp85 to Tva protein (Figures 6B,C, *P* < 0.05). Residues s7, s8, K251, and L252 significantly reduced the percentage of GFP-positive cells (*P* < 0.001), and viral TCID_50_ assays confirmed that the substitution of these single amino acids significantly decreased the viral titers (Figures 6F,G). However, the single amino acid mutation of A256S had no significant effect on the viral titer (Figure 6G, *P* > 0.05).

### Single Amino Acid Mutation, G196A and R198H, Terminated the Replication Ability of the Recombinant Virus

**FIGURE 7:**
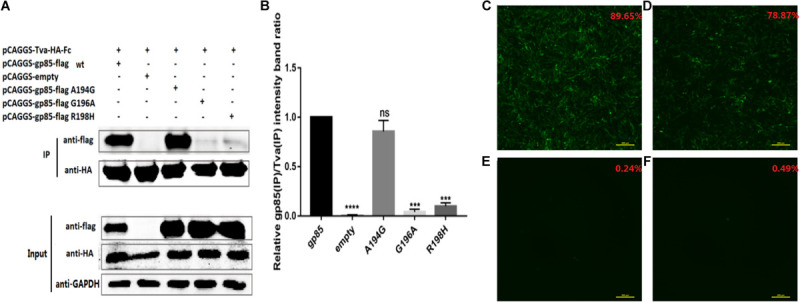
Single amino acid mutation, G196A and R198H, terminated the replication ability of the recombinant virus. **(A)** The interaction of individual amino acid substitution (A194G, G196A, and R198H) in soluble chimeric protein gp85-flag s2 with Tva-HA-Fc, for which the proteins were immunoblotted with anti-flag or anti-HA mAb. **(B)** The level of protein was quantified using Image StudioLiteVer 5.2, and soluble chimeric proteins gp85-flag (IP)/Tva-HA-Fc (IP) were calculated. **(C)** The fluorescence signal of recombinant virus RCASBPGDFX0602-EGFP and the percentage of GFP-positive cells are shown in the upper right corner of each image. **(D)** The fluorescence signal of recombinant virus RCASBPGDFX0602 A194G-EGFP. **(E)** The fluorescence signal of recombinant virus RCASBPGDFX0602 G196A-EGFP. **(F)** The fluorescence signal of recombinant virus RCASBPGDFX0602 R198H-EGFP. Three independent experiments were performed, and data are shown as mean ± SD in triplicate from a representative experiment. Statistical analysis (two-way analysis of variance) was performed using GraphPad Prism 7; ***P < 0.001, ****P < 0.0001, ^*ns*^*P* > 0.05.

Amino acids A194, G196, and R198 in the residues 194–199 of GDFX0602 gp85 differed from those of ev-1 gp85. Also, Co-IP results showed that single amino acids G196 and R198 take part in the interaction, but A194 does not exert any effect therein. Moreover, single amino acids G196A and R198H almost negated the binding of gp85 to Tva (Figures 7A,B). To identify the results of co-IP, we further analyzed the effect of mutants on the replication ability of recombinant viruses. As expected, compared with the DF-1 cells transfected with RCASBPGDFX0602-EGFP (89.65%), the percentage of GFP fluorescence signal in the DF-1 cells transfected with RCASBP GDFX0602 A194G-EGFP (78.87%) was slightly lower (Figures 7C,D), whereas the GFP fluorescence signal was almost not observed in the DF-1 cells transfected with RCASBPGDFX0602 G196A-EGFP (0.24%) and RCASBP GDFX0602 R198H-EGFP (0.49%, Figures 7E,F).

## Discussion

Although ALV-K, a novel subgroup ALV, is endemic in domestic chickens in China (Dong et al., 2015a), its clinical characteristics have not been clearly defined because of its weak pathogenicity and replication ability. However, recent ALV-K isolates with mutations in *pol* gene have shown competitive replication advantages both *in vivo* and *in vitro* (Su et al., 2018b). Therefore, the molecular epidemiology of ALV-K requires more attention. Most ALV-K isolates have endogenous LTR that shows considerable homology with that of endogenous virus ALV-E (Li et al., 2016; Zhao et al., 2018; Lv et al., 2019). The genomic difference between ALV-K and ALV-E was centered in SU encoded by gp85 (Figure 1) because ALV-E does not infect DF-1 (Federspiel et al., 1991); hence, the difference determines the receptor type of virus involved in invading the DF-1 cells. In this study, we used domain replacement strategies for gp85 of ALV-K GDFX0602 and ALV-E ev-1 to demonstrate that the gp85 of ALV-K, especially hr1, is critical for Tva binding.

In other retrovirus studies, the SU of retroviruses that bind to cell receptors can be categorized into two types: one concentrated in the region where SU is highly variable, as in mouse leukemia virus and ALV-A, -B, -C, -D, and -E (Battini et al., 1995); and the other composed of discontinuous, multiple variable, and conserved regions of human immunodeficiency virus gp120 that binds to CD4 (Cordonnier et al., 1989; Kwong et al., 1998). Similarly, the region of equine infectious anemia virus that binds to cell receptors is the discontinuous sequence of gp90 (Sun et al., 2008). The amino acid residues of ALV-A SU that bind to Tva are located in hr2 (Rong et al., 1997b; Damico et al., 1999). Our results showed that both hr1 and hr2 can affect the replication of ALV-K in DF-1 cells and affect the interaction between SU and Tva. On the other hand, the amino acid residues of SU that binds to Tva are localized at residues 194–198 and 206–216 of hr1 (Figures 4B,C), residues 251–256 between hr1and hr2, and residues 269–280 of hr2 (Figures 5B,C).

Previously, the deletion of six amino acids in hr1 of ALV-A SU reduced the infection titer, but SU could interact with Tva (Holmen and Federspiel, 2000). Similarly, the deletion of 13 amino acids in hr1 of ALV-B SU did not affect binding with Tvb but rendered Env more resistant to fusion activation at a low pH (Rose et al., 2007). Our study also showed similar results: substitution of some amino acid residues (s7, s8, and K251E) significantly reduced the percentage of GFP-positive cells and viral infection titer (Figures 6F,G), but the binding affinity of recombinant soluble proteins (gp85 s7, gp85 s8, gp85 K251E) and Tva protein receptor was not significantly affected (Figures 6B,C, *P* > 0.05). The amino acids in residues gp85 s1, gp85 L252, and gp85 A256 enhanced the binding affinity of gp85 to Tva protein, and the difference was significant (Figures 4B,C, 6B,C, *P* > 0.05), but the effect on the level of virus replication was insignificant (Figures 4D,E, 6F). Therefore, the mutations in amino acid residues s1, s7, s8, and s9 (aa189–193 and aa237–256) do not affect the binding affinity of gp85 to Tva, but the percentage of GFP-positive cells and the virus titer of recombinant ALV-K is significantly reduced (*P* < 0.0001). Of course, the cytotoxic effect of the recombinant glycoproteins on the cells may be one reason that affects the percentage of GFP-positive cells and the virus titer (Federspiel, 2019). In addition, the western blotting gray levels of recombinant gp85 s6 (aa217–221) and K251 were slightly lower than those of gp85 (positive control), but the difference was not statistically significant (Figures 4C, 6C, *P* > 0.05). We therefore assumed that aa217–221 and K251E may also affect the binding affinity of gp85 to Tva, although this possibility requires further demonstration.

Previous studies have identified hr1 and hr2 as the principal binding domains between the viral glycoprotein trimer and the host protein receptor (Dorner and Coffin, 1986; Bova et al., 1988; Holmen et al., 2001; Federspiel, 2019). In addition, previous studies have found that vr3 affected the binding of ALV-A capsule membrane protein to Tva (Melder et al., 2003), but our results show that the mutation of vr3 has no significant effect on ALV-K gp85 binding to Tva (Figures 6D,E). Enveloped viruses use several viral encoded glycoproteins to mediate the binding of the viral and host cell membranes. The single N-linked glycosylation site in the SU domain of EnvA is critical for binding between EnvA and its receptor Tva (Delos et al., 2002). Our results also showed that the changes in a single amino acid (G196A or R198H) can negate the binding of ALV-K gp85 to Tva receptor (Figures 7A,B), which was further corroborated by the fluorescence signal of the recombinant virus (Figures 7E,F).

Avian leukosis viruses replicate with extremely high mutation rates because of poor fidelity during reverse transcription and replication; retroviruses therefore exhibit great genetic diversity that allows a viral population to rapidly adapt to different environments and exhibit resistance to host immune responses and antiviral drugs (Dong et al., 2017b). During evolution, the virus is subjected to external selection pressures and may change the way that it invades cells by changing a few amino acids (Yin et al., 2019). Avian sarcoma leucosis virus in the presence of a competitor to the subgroup A Tva receptor, SUA-rIgG immunoadhesin, evolves to use other receptors (Melder et al., 2003). In addition, the selected mutant virus RCASBP(A)Δ155–160 modestly expanded the use of Tvb and Tvc receptors and possibly other cell surface proteins while maintaining the binding affinity to Tva (Munguia and Federspiel, 2019). Our results showed that hr1 (aa194–198 and aa206–216) and hr2 (aa251–256 and aa269–280) regions bind to Tva; hence, hr1 and hr2 could be changed under selection pressure in the presence of immune adhesins. Therefore, the evolution direction of hr of ALV-K in the presence of SUA-rIgG immune adhesins would be an interesting field to explore.

## Conclusion

To identify the functional determinants of ALV-K envelope protein that binds to its receptor Tva, the continuous, segment-by-segment substitution of the gp85-encoded surface glycoprotein (SU) of ALV-K GDFX0602 with ALV-E ev-1 was performed. A series of recombinant viruses with RCASBP vector as their skeletons were created for the virus infection experiment, and a series of chimeric soluble gp85 proteins were expressed for co-IP experiments. Our results showed that all s1–s13 residues decreased the infection titer of the recombinant virus, wherein s2–s7 (aa194–243), s9 (aa251–256), G196A, R198H, K251E, and L252I almost negated the infection affinity of the recombinant virus. Furthermore, residues s2 (aa194–198), s4, and s5 (aa206–216) in hr1 and s9 (aa251–256), s12, and s13 (aa269–280) in hr2 played a key role in gp85 binding to Tva, and single amino acid mutations G196A and R198H almost negated their binding. Furthermore, the results of the recombinant virus fluorescence signal further confirmed that G196A and R198H played a key role in the binding of ALV-K gp85 to Tva. Our study identified the key amino acids involved in binding of ALV-K SU to Tva, which may help to further clarify the ALV-K infection mechanism.

## Data Availability Statement

The original contributions presented in the study are included in the article/supplementary material, further inquiries can be directed to the corresponding author/s.

## Author Contributions

JC and WC participated in the design of the study, performed the experiments, collected and analyzed data, and drafted the manuscript. JL constructs a series of RCASBP-based recombinant viruses and pCAGGS-based recombinant gp85 proteins. LL and PL provided assistance in flow cytometry and co-IP experiments. YX performed the statistical analysis. WC participated the design and coordination of the study. All authors contributed to the article and approved the submitted version.

## Conflict of Interest

The authors declare that the research was conducted in the absence of any commercial or financial relationships that could be construed as a potential conflict of interest.

## References

[B1] Adkins H. B. Brojatsch J. Naughton J. Rolls M. M. Pesola J. M. Young J. A. (1997). Identification of a cellular receptor for subgroup E avian leukosis virus. *Proc. Natl. Acad. Sci. U S A.* 94 11617–11622. 10.1073/pnas.94.21.11617 9326659 PMC23555

[B2] Battini J. L. Danos O. Heard J. M. (1995). Receptor-binding domain of murine leukemia virus envelope glycoproteins. *J. Virol.* 69 713–719. 10.1128/JVI.69.2.713-719 7815534 PMC188633

[B3] Bova C. A. Olsen J. C. Swanstrom R. (1988). The avian retrovirus env gene family: molecular analysis of host range and antigenic variants. *J. Virol.* 62 75–83. 10.1128/JVI.62.1.75-83 2824857 PMC250503

[B4] Cordonnier A. Rivière Y. Montagnier L. Emerman M. (1989). Effects of mutations in hyperconserved regions of the extracellular glycoprotein of human immunodeficiency virus type 1 on receptor binding. *J. Virol.* 63 4464–4468. 10.1128/JVI.63.10.4464-4468 2550679 PMC251071

[B5] Cui N. Su S. Chen Z. Zhao X. Cui Z. (2014). Genomic sequence analysis and biological characteristics of a rescued clone of avian leukosis virus strain JS11C1, isolated from indigenous chickens. *J.Gen. Virol.* 95:2512. 10.1099/vir.0.067264-0 25009192

[B6] Damico R. Rong L. Bates P. (1999). Substitutions in the receptor-binding domain of the avian sarcoma and leukosis virus envelope uncouple receptor-triggered structural rearrangements in the surface and transmembrane subunits. *J. Virol.* 73 3087–3094. 10.1128/JVI.73.4.3087-3094 10074159 PMC104069

[B7] Delos S. E. Burdick M. J. White J. M. (2002). A single glycosylation site within the receptor-binding domain of the avian sarcoma/leukosis virus glycoprotein is critical for receptor binding. *Virology* 294 354–363. 10.1006/viro.2001.1339 12009877

[B8] Dong X. Meng F. Hu T. Ju S. Li Y. Sun P. (2017b). Dynamic co-evolution and interaction of avian leukosis virus genetic variants and host immune responses. *Front. Microbiol.* 8:1168. 10.3389/fmicb.2017.01168 28694798 PMC5483431

[B9] Dong X. Zhao P. Xu B. Fan J. Meng F. Sun P. (2015a). Avian leukosis virus in indigenous chicken breeds. *China.Emerg.Microbes. Infect.* 4:e76. 10.1038/emi.2015.76 26714782 PMC4715165

[B10] Dorner A. J. Coffin J. M. (1986). Determinants for receptor interaction and cell killing on the avian retrovirus glycoprotein gp85. *Cell* 45 365–374. 10.1016/0092-8674(86)90322-3 3009025

[B11] Fadly A. M. Smith E. J. (1999). Isolation and some characteristics of a subgroup J-like avian leukosis virus associated with myeloid leukosis in meat-type chickens in the united states. *Avian Dis.* 43 391–400. 10.2307/1592636 10494407

[B12] Federspiel M. J. (2019). Reverse engineering provides insights on the evolution of subgroups a to e avian sarcoma and leukosis virus receptor specificity. *Viruses* 11 1–25. 10.3390/v11060497 31151254 PMC6630264

[B13] Federspiel M. J. Crittenden L. B. Provencher L. P. Hughes S. H. (1991). Experimentally introduced defective endogenous proviruses are highly expressed in chickens. *J. Virol.* 65 313–319. 10.1128/JVI.65.1.313-319 1845892 PMC240519

[B14] Guan X. Zhang Y. Yu M. Ren C. Gao Y. Yun B. (2017). Residues 28 to 39 of the extracellular loop 1 of chicken Na+/H+ exchanger type i mediate cell binding and entry of subgroup j avian leukosis virus. *J. Virol.* 92 1617–1627. 10.1128/JVI.01627-17 29070685 PMC5730758

[B15] Holmen S. L. Federspiel M. J. (2000). Selection of a subgroup a avian leukosis virus [ALV(A)] envelope resistant to soluble ALV(A) surface glycoprotein. *Virology* 273 364–373. 10.1006/viro.2000.0424 10915607

[B16] Holmen S. L. Melder D. C. Federspiel M. J. (2001). Identification of key residues in subgroup a avian leukosis virus envelope determining receptor binding affinity and infectivity of cells expressing chicken or quail Tva receptor. *J. Virol.* 75 726–737. 10.1128/JVI.75.2.726-737 11134286 PMC113969

[B17] Klucking S. Young J. A. T. (2004). Amino acid residues Tyr-67, Asn-72, and Asp-73 of the TVB receptor are important for subgroup E avian sarcoma and leukosis virus interaction. *Virology* 318 371–380. 10.1016/j.virol.2003.09.024 14972562

[B18] Knauss D. J. Young J. A. T. (2002). A fifteen-amino-acid TVB peptide serves as a minimal soluble receptor for subgroup b avian leukosis and sarcoma viruses. *J. Virol.* 76 5404–5410. 10.1128/jvi.76.11.5404-5410.2002 11991969 PMC137033

[B19] Koslová A. Kuèerová D. Reinišová M. Geryk J. Trefil P. Hejnar J. (2018). Genetic resistance to avian leukosis viruses induced by crispr/cas9 editing of specific receptor genes in chicken cells. *Viruses* 10:605. 10.3390/v10110605 30400152 PMC6266994

[B20] Kucerová D. Plachy J. Reinisová M. Senigl F. Trejbalová K. Geryk J. (2013). Nonconserved tryptophan 38 of the cell surface receptor for subgroup J avian leukosis virus discriminates sensitive from resistant avian species. *J. Virol.* 87 8399–8407. 10.1128/JVI.03180-12 23698309 PMC3719790

[B21] Kwong P. D. Wyatt R. Robinson J. Sweet R. W. Sodroski J. Hendrickson W. A. (1998). Structure of an HIV gp120 envelope glycoprotein in complex with the CD4 receptor and a neutralizing human antibody. *Nature* 393 648–659. 10.1038/31405 9641677 PMC5629912

[B22] Li X. Lin W. Chang S. Zhao P. Zhang X. Liu Y. (2016). Isolation, identification and evolution analysis of a novel subgroup of avian leukosis virus isolated from a local chinese yellow broiler in south china. *Arch. Virol.* 161 2717–2725. 10.1007/s00705-016-2965-x 27422398

[B23] Lv L. Li T. Hu M. Deng J. Liu Y. Xie Q. (2019). A recombination efficiently increases the pathogenesis of the novel K subgroup of avian leukosis virus. *Vet. Microbiol.* 231 214–217. 10.1128/JVI.73.4.3087-3094 30955812

[B24] Melder D. C. Pankratz V. S. Federspiel M. J. (2003). Evolutionary pressure of a receptor competitor selects different subgroup a avian leukosis virus escape variants with altered receptor interactions. *J. Virol.* 77 10504–10514. 10.1128/jvi.77.19.10504-10514 12970435 PMC228527

[B25] Munguia A. Federspiel M. J. (2019). Avian sarcoma and leukosis virus envelope glycoproteins evolve to broaden receptor usage under pressure from entry competitors. *Viruses* 11:519. 10.3390/v11060519 31195660 PMC6630762

[B26] Pøikryl D. Plachı J. Kuèerová D. Koslová A. Hejnar J. (2019). The novel avian leukosis virus subgroup K shares its cellular chicken receptor with subgroup A. *J. Virol.* 93 e519–e580. 10.1128/JVI.00580-19 31217247 PMC6694804

[B27] Rong L. Bates P. (1995a). Analysis of the subgroup A avian sarcoma and leukosis virus receptor: the 40-residue, cysteine-rich, low-density lipoprotein receptor repeat motif of Tva is sufficient to mediate viral entry. *J. Virol.* 69 4847–4853. 10.1128/JVI.69.8.4847-4853 7609052 PMC189298

[B28] Rose B. A. James B. John A. T. Y. (2007). The hr1 and fusion peptide regions of the subgroup B avian sarcoma and leukosis virus envelope glycoprotein influence low pH-Dependent membrane fusion. *Plos One* 2:e171. 10.1371/journal.pone.0000171 17245447 PMC1764858

[B29] Smith J. G. Cunningham J. M. (2007). Receptor-induced thiolate couples env activation to retrovirus fusion and infection. *PLoS Pathog.* 3:e198. 10.1371/journal.ppat.0030198 18260686 PMC2151085

[B30] Rong L. Edinger A. Bates P. (1997b). Role of basic residues in the subgroup-determining region of the subgroup A avian sarcoma and leukosis virus envelope in receptor binding and infection. *J. Virol.* 71:3458 10.1128/JVI.71.5.3458-3465 PMC191492 9094617

[B31] Su Q. Li Y. Li W. Cui S. Tian S. Cui Z. (2018a). Molecular characteristics of avian leukosis viruses isolated from indigenous chicken breeds in China. *Poult. Sci.* 97 2917–2925. 10.3382/ps/pex367 29800289

[B32] Su Q. Li Y. Cui Z. Chang S. Zhao P. (2018b). The emerging novel avian leukosis virus with mutations in the pol gene shows competitive replication advantages both in vivo and in vitro. *Emerg. Microbes. Infect.* 7:117. 10.1038/s41426-018-0111-4 29946141 PMC6018675

[B33] Sun C. Zhang B. Jin J. Montelaro R. C. (2008). Binding of equine infectious anemia virus to the equine lentivirus receptor-1 is mediated by complex discontinuous sequences in the viral envelope gp90 protein. *J. Gen. Virol.* 89 2011–2019. 10.1099/vir.0.83646-0.28 18632973

[B34] Sun C. Zhang B. Jin J. Montelaro R. C. (2008). Binding of equine infectious anemia virus to the equine lentivirus receptor-1 is mediated by complex discontinuous sequences in the viral envelope gp90 protein. *J. Virol.* 89 2011–2019. 10.1099/vir.0.83646-0 18632973

[B35] Taplitz R. Coffin J. M. (1997). Selection of an avian retrovirus mutant with extended receptor usage. *J. Virol.* 71 7814–7819. 10.1128/JVI.71.10.7814-7819.1997 9311868 PMC192135

[B36] Yin X. Melder D. C. Payne W. S. Dodgson J. B. Federspiel M. J. (2019). Mutations in both the surface and transmembrane envelope glycoproteins of the rav-2 subgroup b avian sarcoma and leukosis virus are required to escape the antiviral effect of a secreted form of the tvbs3 receptor. *Viruses* 11:500. 10.3390/v11060500 31159208 PMC6630269

[B37] Zhao Z. Rao M. Liao M. Cao W. (2018). Phylogenetic analysis and pathogenicity assessment of the emerging recombinant subgroup K of avian leukosis virus in south china. *Viruses* 10:194. 10.3390/v10040194 29652854 PMC5923488

